# Severe macrophage activation syndrome. Is there a causative role for a homozygous A91V mutation in the perforin gene?

**DOI:** 10.1186/1546-0096-13-S1-P20

**Published:** 2015-09-28

**Authors:** H Girschick, R Rossi, U Kölsch, S Ammann, P Lohse, H Morbach, H von Bernuth, S Ehl

**Affiliations:** 1 Vivantes Childrens Hospital Berlin, Pediatrics, Berlin, Germany

## 

A 16 year old lebanese girl with consanguinous parents presented with a severe “abdominal” sepsis supposedly resulting from an infected vaginal tampon (ESBL E.coli). She had been healthy before. She developed severe hepatic functional disorder, infarction of the spleen, cardiovascular and renal insufficiency, as well as anemia and thrombocytopenia. Macrophage activation syndrome was diagnosed subsequently and systemic glucocorticoid treatment initiated. The girl recovered clinically. Of note, she developed severe cushingoid syndrome. Due to limited compliance she discontinued all anti-inflammatory medication (nsaids, gc and ciclosporin) 4 weeks later. The following 18 months inflammatory parameters were persistantly elevated (ESR>100mm/h). Familial mediteranean fever was excluded. Genetic analysis revealed a homozygous perforin I gene mutation 91 (GCG) -> Valin (GTG)/.pAla91Val-/A91V in exon 2. Familial hemophagocytic lymphohistiocytosis type II was discussed as a potential diagnosis. Perforin expression was diminshed to about 50% in NK-cells, however functional NK-cell cytotoxicity was in the lower normal range, considered not impaired.

On the basis of these findings, we want to discuss the role of the homozygous A91V perforin mutation for the initiation or perpetuation of a life-threatening macrophage activation syndrome, in addition to the further management of the patient.

## Consent to publish

Written informed consent for publication of their clinical details was obtained from the patient/parent/guardian/relative of the patient.

